# CMRSegTools: an Osirix plugin for myocardial infarct sizing on DE-CMR images

**DOI:** 10.1186/1532-429X-16-S1-P204

**Published:** 2014-01-16

**Authors:** Magalie Viallon, Joel Spaltenstein, Charles de Bourguignon, Coralie Vandroux, Olivier Bernard, Loic Belle, Patrick Clarysse, Pierre Croisille

**Affiliations:** 1 CREATIS UMR CNRS 5220 INSERM U1044, Université de Lyon, Lyon, France; 2 Spaltenstein Natural Image, Geneva, Switzerland; 3 Cardiology, CH Annecy, Annecy, France; 4 Radiology, CHU Saint-Etienne, Université J. Monnet, saint-Etienne, France

## Background

CMR is an established method for the diagnosis and assessment of myocardial infarction in routine exams. In addition, there has been a growing interest in using infarct size as a surrogate endpoint for clinical trials assessing the efficacy of acute MI reperfusion treatments and MI size is reported to be a stronger predictor of outcome than LVEF and LV volumes[1].Various methods have been proposed for infarct size (IS) measurement without reference standard method and side-by-side comparison among them. The goal was to develop a user-friendly cardiac segmentation toolbox implemented as a plugin in the open-source OsiriX software with a set a commonly proposed semi-automatic algorithms

## Methods

Contour segmentation was implemented using a fast automated segmentation framework (B-spline Explicit Active Surfaces[2]) that could be corrected or manually performed. Several semiautomatic methods were implemented from simple image intensity threshold based on 1) histogram, or 2) equal to × standard deviation (xSD) above the mean of remote normal myocardial intensity 3) full-width at half-maximum (FWHM) with 2D/3D and maximum or region based variants. More sophisticated algorithms were also implemented including 4) the Gaussian Mixture Model (GMM) and 5) the method proposed by Hsu that assign a weighting to each voxel depending on its image intensity or use a combination of methods along with regional feature analysis. Performance of methods was 1) evaluated for precision and accuracy on a realistic phantom based on real datasets with varying contrast to noise ratio (CNR) between infarct aera and normal myocardium and 2) compared in-vivo on DE-CMR images in post-AMI patients.

## Results

**Figure 1:**
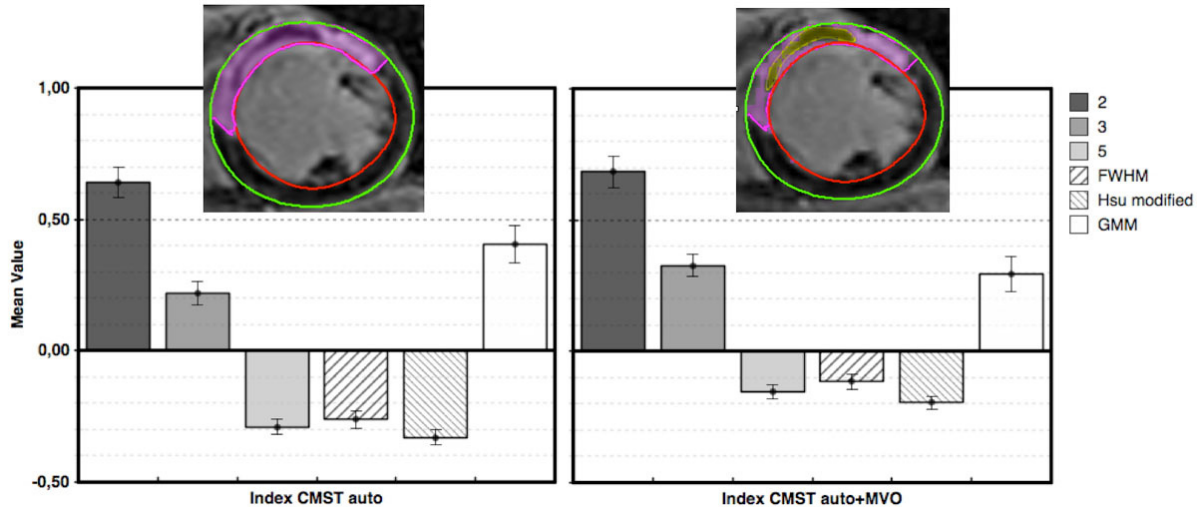
**(Left) Performance of semi-automatic method on patients without any user interaction, and (Right) after inclusion of MVO in the measures**. Mean values of the defined relative error obtained on 10 patient dataset and standard error of mean to mean bar, considering all slices from a 3D series of LGE images. The y = 0 ordinate is the line of perfect concordance between the expert and the semi-automatic method.

**Figure 2:**
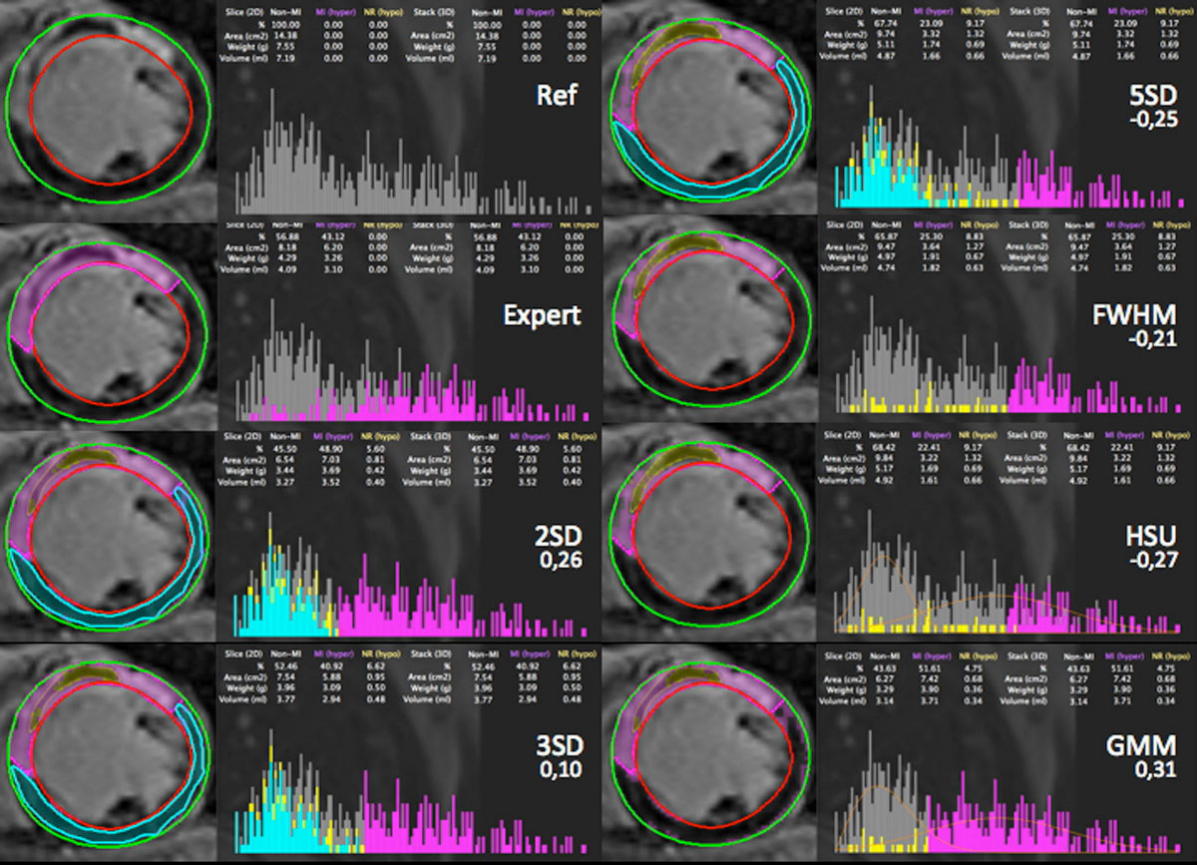
**The error to expert segmentation is given below the name of the method on the image and is calculated as: error=(Aera_MI(method)-Area_MI(reference expert))/Aera_MI(reference expert): =: accurate results, >0: over-estimation, <0: under-estimation**. The expert segmentation is given for reference as a pink line in each segmented images. all pixels within the reference ROI used for the xSD method are categorized in cyan blue, MVO as yellow.

When evaluated on the phantom study, accuracy of all algorithms reached 100% when CNR>7. For low CNR values, whereas xSD and FWHM appeared more robust, GMM illustrated its intrinsically higher sensitivity to the noise level. Figure 1 shows the overall performance of implemented technique. Figure 2 shows results obtained in-vivo in a patient with a large infract including a micro-vascular obstruction (MVO) lesion. Whereas 2SD and 3SD tends to overestimate IS, 5SD and FWHM are close to the expert results, when MVO lesions, if present, are added to the segmented lesions.

## Conclusions

CMRSegTools brings in a user friendly interface integrated as a plugin in the widely available advanced open-source OsiriX dicom workstation, a toolbox allowing to calculate, display and interactively analyze myocardial lesions and especially myocardial infarct lesions. CMRSegTools offers a fast, real-time and integrated process and advanced published algorithms for quantification purposes in CMR through an ergonomic interface. It can be proposed for newly available methods, as a plateform for algorithm performance comparison and validation.
